# Wireless power transfer system for deep-implanted biomedical devices

**DOI:** 10.1038/s41598-022-18000-6

**Published:** 2022-08-11

**Authors:** Amjad Iqbal, Penchala Reddy Sura, Muath Al-Hasan, Ismail Ben Mabrouk, Tayeb A. Denidni

**Affiliations:** 1 Institut National de la Recherche Scientifique (INRS), Montréal, QC H5A1K6 Canada; 2 Department of Network and Communications Engineering, Al Ain University, Al Ain, 64141 United Arab Emirates; 3 Department of ECE, Visvodaya Engineering College, Kavali, 524201 India; 4 Department of Engineering, Durham University, Durham, DH1 3LE UK

**Keywords:** Electrical and electronic engineering, Medical research

## Abstract

In this paper, a dual-band implantable rectenna is proposed for recharging and operating biomedical implantable devices at 0.915 and 2.45 GHz. The rectenna system consists of a compact dual-band antenna based on a meandered-resonator as well as efficient dual-band rectifier circuit. Both components (antenna and rectifier) are integrated inside a capsule device to simulate and experimentally validate the rectenna. The antenna occupies lower volume ($5 \times 5.25 \times 0.25$ $\hbox {mm}^{3}$), where compactness is achieved using meandered geometry and a slotted ground plane. It maintains quasi-omnidirectional radiation patterns and peak realized gains of −22.1 dBi (915 MHz) and −19.6 dBi (2.45 GHz); thus, its capability is enhanced to harvest the ambient energy from multiple directions. Moreover, a dual-band rectifier is designed using a dual-branch matching network (an L-matching network and open-circuited stub in each branch) with a radio frequency (RF) to direct current (DC) conversion efficiency of 79.9% for the input power of 1 dBm (lower band: 0.915 GHz) and 72.8% for the input power of 3 dBm (upper band: 2.45 GHz). To validate the concept of the rectenna, the implantable antenna and rectifier are fabricated and attached together inside a capsule device, with the measured results verifying the simulated responses. The proposed rectenna efficiently rectifies two RF signals and effectively superimposes on a single load, thus, providing a distinct advantage compared to single-band rectennas. To the best of the authors’ knowledge, this is the first-ever implantable rectenna to perform dual-band RF signal rectification.

## Introduction

**Figure 1:**
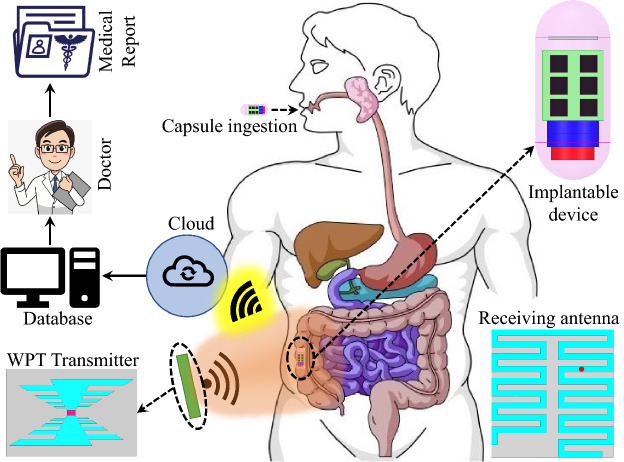
Generalized wireless power transfer (WPT) system for deep-implanted biomedical devices.

The use of biomedical implantable devices (BIDs) for healthcare applications, such as drug delivery and bio-telemetry, is no longer just science fiction^1–3^. Recently, major developments have been made in using BIDs for deep brain stimulation^4^, cardiac pacemakers^5^, glucose monitoring^6^, intra-oral tongue drive systems^7^, and capsule endoscopy^8^, among others. Since the beginning, primary batteries and wired power sources have been used in these implants. The lifetime of the primary batteries, however, is limited and must be replaced after a single use. Moreover, operations are required to replace these batteries, which is inconvenient, costly, and painful for the patient. Furthermore, wired power sources are unsafe and may cause further infections and diseases. To tackle these challenges and limitations, many methods have been developed to harvest energy from different sources, such as light, heat, sound, pressure, and vibration^9–11^. Moreover, ambient radio frequency (RF) sources can also be harvested to drive the electronic components and recharge the batteries inside the implant^12^. As a result, RF sources are preferable for driving biomedical implants^13^. Harvesting these sources, however, is a challenging task as the amplitude of the available signals is quite low and the power required for driving the biomedical implants is high. In fact, in implants where high driving power is required, a specialized wireless power transmitter can be used as an RF source^14^. The power transmitted from the specialized transmitter source through the medium of air is known as wireless power transfer (WPT) (Fig. 1), which is dependent on the coupling of the transmitter and receiver^15^. Compared to conventional ambient RF sources, WPT has become a suitable candidate to charge and drive the implants due to the transmitter’s controlled nature (direction of power can be controlled)^16^. The efficiency of the WPT system is dependent on the efficiency of both the rectenna and power transmission.

The WPT system consists of a rectenna and a power source (a WPT transmitter). The rectenna consists of two parts: (1) rectifier and (2) antenna. The antenna receives RF signals and the rectifier circuit converts it into usable direct current (DC) power, which can be stored in the batteries or used to drive the implants^14,17^. Since most of the commercial WPT transmitters have a fixed signal strength, the receiver side (antenna and rectifier) should be designed in such a way to harvest the minimum signal available with high efficiency. Consequently, an efficient rectifier is necessary for harvesting a wide range of available source with a high efficiency.

Recently, several rectenna systems have been developed for harvesting the sources emitted from the WPT transmitters. In^18^, a millimeter-sized rectenna is designed at 915 MHz for a deep brain stimulation device. A low-profile antenna and rectifier circuit are integrated to harvest the radiated energy from the source. The reported rectifier has a maximum RF-to-DC conversion efficiency of 61.53% at an input power of 5 dBm, however, it suffers from narrow bandwidth. In^19^, a triple-band (402, 433, and 2450 MHz) implantable antenna with overall size of $10\times 10\times 2.54$ $\hbox {mm}^{3}$ for bio-telemetry devices is discussed. Then the same antenna is connected to a rectifier at 433 MHz to accomplish wireless powering. It has an RF-to-DC conversion efficiency of 86% at an input power of 11 dBm (load = 5 $\hbox {K}\Omega$). In^20^, a battery-less cardiac pacing is reported, using an external WPT transmitter as a power source, an implantable antenna as a receiving antenna, and a rectifier as an RF-to-DC conversion unit. The rectifier has an RF-to-DC conversion efficiency of 57% at an input power of 0 dBm. In^21^, a miniaturized deep-body implantable rectenna is designed at 673 MHz for use in cardiac pacemakers. The rectifier circuit has an RF-to-DC conversion efficiency of 40% at an input power of −20 dBm. A dual-band implantable antenna is designed in^22^, using a 402 MHz frequency band for bio-telemetry applications and a 915 MHz frequency band for energy harvesting. One-diode and two-diode topologies are used for the design of a rectifier. At an input power of −5 dBm, it achieves RF-to-DC conversion efficiencies of 51.7% and 40.4% in one-diode topology and two-diode topology rectifiers, respectively. In^23^, a WPT system is designed at 915 MHz, with the external patch acting as an RF source. Moreover, a rectifier integrated antenna acts as a receiving unit that converts RF signals to useful DC power. The rectifier circuit has an RF-to-DC efficiency of 59.8% at an input power of 30 dBm. In^24^, a circularly polarized implantable antenna for far-field wireless powering is proposed. The rectifier circuit has an RF-to-DC efficiency of 44% at an input power of −10 dBm. The authors of^25^ details the design of a far-field WPTs for skin-implanted devices, where an RF-to-DC efficiency of 42% is achieved at −10 dBm. Moreover, the power transmission efficiency (PTE) is enhanced by adding a high-permittivity dielectric on the body. The transmission coefficient ($\hbox {S}_{21}$) of the WPT system is improved from −54.1 to −41.3 dB with the addition of the dielectric material. In^26^, a near-field WPT system is designed for scalp-implanted biomedical devices, using a dual-band antenna (915 MHz and 1900 MHz), a single band rectifier (1900 MHz), and a single-band WPT transmitter (1900 MHz). The maximum RF-to-DC conversion efficiency of 82% is achieved at an input power of 2 dBm. A PTE of 0.007% is achieved by placing the WPT 20 mm away from the implantable antenna. Similarly, a mid-field WPT system is designed in^27^, consisting of a quad-band (0.403 GHz, 0.915 GHz, 1.47 GHz, and 2.4 GHz) implantable antenna, a single-band (1.47 GHz) rectifier and a single-band (1.47 GHz) WPT transmitter. The designed rectifier has a maximum RF-to-DC conversion efficiency of 90% at 2 dBm. Furthermore, the WPT system has PTE of 0.67% at 1.47 GHz. In all of the above designs, the implantable antenna size is large, which, in turn, increases the size of the implant. In fact, since an antenna is an important part of the rectenna circuit, the size of the implantable device is mainly dependent on the size of the antenna. Therefore, a small-sized implantable antenna is required for compacting the BIDs. Moreover, all of the above rectifiers can only operate at one frequency band, and, owing to the complex circuitry of current implants, high amount of power are required for their operations. Therefore, multi-band WPT systems are an excellent choice for wireless powering when compared to single-band WPT systems^28^.

In this paper, a dual-band WPT system is designed, where a wideband log periodic antenna acts as a source for the implantable rectenna. Initially, each part of the WPT system is individually designed and experimentally tested. Then, the PTE is analyzed, and a matching layer is used to enhance it. Finally, the rectifier is connected to the implantable antenna. As a proof, the log periodic antenna (the WPT transmitter) is sourced with an RF signal generator, the integrated-implantable antenna is placed inside minced pork meat and the received voltage is observed on digital multimeter (DMM). The proposed dual-band rectenna is an excellent choice for future implants due to its compact dimensions, human safety analysis, dual-band RF signals rectification, and high RF-to-DC conversion efficiency. Moreover, to the best of the authors’ knowledge, this is the first-ever implantable rectenna to perform dual-band RF signal rectification.

**Figure 2:**
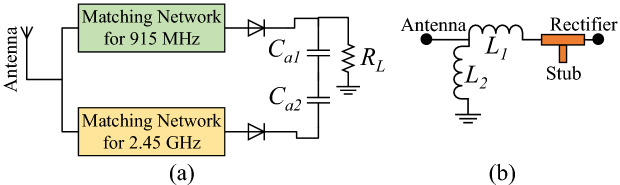
Dual-band rectifier (**a**) proposed topology ($C_{a1}$ = $C_{a2}$ 18 pF and $R_{L}$ = 10 K$\Omega$), and (**b**) matching network ($L_{1l}$ = 23 nH, $L_{2l}$ = 40 nH, $L_{1u} = 4.3$ nH and $L_{2u} = 0.2$ nH).

## Dual-band rectifier design

### Matching network and series-diode design

A rectifier circuit has the ability to obtain useful DC power from the RF sources. Figure 2 shows general topology of the proposed dual-band (915 MHz and 2.45 GHz) rectifier. An optimal frequency is needed to realize an efficient WPT system^29–31^. These frequency bands (ISM bands of 915 MHz and 2.45 GHz) are selected because they do not require license. Initially, the optimal frequency is set to be 915 MHz. Then, 2.45 GHz is also chosen because it is license free band. The optimal frequency is derived using the Equation (1)^32^.

1 $$\begin{aligned} f_{opt} = \dfrac{1}{2\pi }\sqrt{\dfrac{c\sqrt{\epsilon _{r0}}}{d\tau (\epsilon _{r0}-\epsilon _{\infty })}} \end{aligned}$$

where, $f_{opt}$ is the optimal frequency, $\epsilon _{r0}$ is the static permittivity, *c* is the speed of light, $\tau$ is the relaxation time constant and $\epsilon _{\infty }$ is the permittivity at the frequency where $\omega \tau \ge$1^33^. Using Equation (1), the optimum frequency is set to be 915 MHz. Later, the 2.45 GHz frequency band is chosen because it is a license free band.

This design comprises a complex matching network, a rectifying topology, an output filter, and a power management unit or load. Unlike a traditional single-band rectifier, the proposed topology uses two branches of matching networks. The matching circuit between the antenna and rectifier is important, as it determines the amount of power delivered from the antenna to the rectifier. The upper branch matches the lower-frequency ISM band (915 MHz) signal of the antenna with the rectifier, and the lower branch matches the higher-frequency ISM band (2.45 GHz) signal of the antenna with the rectifier. Considering that the complex and non-linear load of the rectifier changes with the frequency, a complex matching network is required. Therefore, the matching network consists of an L-section matching network and an open-circuited stub, as shown in Fig. 2b. The initial values of the two inductors of the L-section matching network are calculated using the following relations^34^:

2 $$\begin{aligned}&L_{1 (l,u)} =\dfrac{1}{\omega _{o}^2{X_{in}}}-L_{2}\bigg (\dfrac{R_{in}}{R_{in}-R_{ant}}\bigg ) \end{aligned}$$

3 $$\begin{aligned}&\quad L_{2 (l,u)}=\bigg (\dfrac{Q}{\omega _{o}R_{in}}\bigg )-{X_{in}} \end{aligned}$$

where $L_{1 (l,u)}$ and $L_{2 (l,u)}$ are the inductances of the matching network, $\omega$ is the angular frequency of the incoming signal, $R_{in}$ is the input resistance of the rectifier, $R_{ant}$ is the resistance of the implantable antenna, *Q* is the quality factor that depends on the bandwidth, and $X_{in}$ is the input reactance of the rectifier. It is worth mentioning that $L_{2 (l,u)}$ as a part of the L-section matching network is also used for DC return path. The values of input resistance ($R_{in}$) and input reactance ($X_{in}$) of the rectifier can be found by simulating the circuit excluding the L-section matching network.

**Figure 3:**
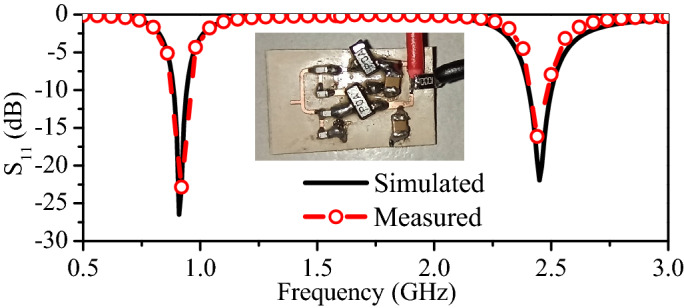
$\hbox {S}_{11}$ (measured and simulated) of the dual-band rectifier.

**Figure 4:**
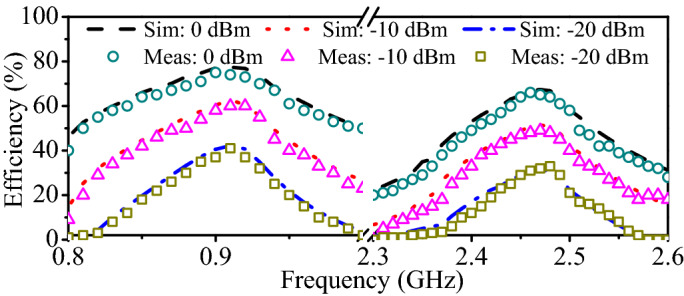
RF-to-DC efficiency at different input powers (0 dBm, −10 dBm, −15 dBm) against operating frequency.

The final values of the inductances, along with the open-circuited stub, are implemented in Keysight Advanced Design System (ADS). The simulations are performed based on two objectives: (1) optimum matching between the source and rectifier and (2) maximum RF-to-DC conversion efficiency at a wide range of input power. The lumped components are connected with one another using microstrip traces. RF Schottky diodes model HSMS-2850 are selected because of their fast switching and lower operating voltage. Initially, the ideal circuit elements of the ADS are used for optimization, but, they are ultimately replaced with muRata components in the ADS library. The optimized schematic of the proposed rectifier is designed on a Rogers RO3010 material ($\epsilon _{r}$ = 10.2) with a thickness of 0.6 mm, as shown in Fig. 3. The simulated $\hbox {S}_{11}$ of the rectifier is compared with measured results in Fig. 3. It can be observed that the proposed rectifier operates at two frequencies (915 MHz and 2.45 GHz). The simulated $\hbox {S}_{11}$ has a fractional bandwidth (FBW) of 7.7% and 5.3% at 915 MHz and 2.45 GHz, respectively. Moreover, the measured FBWs are 7.72% and 5.24% at 915 MHz and 2.45 GHz, respectively.

**Figure 5:**
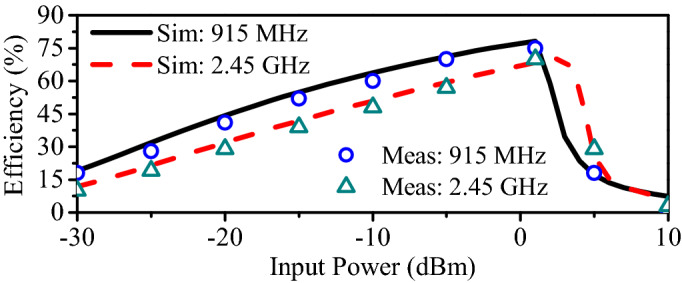
RF-to-DC efficiency at 915 MHz and 2.45 GHz when the load resistance is 10 $\hbox {K}\Omega$.

### RF-to-DC efficiency of the rectifier

Matching networks are designed so as to maximize the available power for the rectifying section. It is worth mentioning that the matching network minimize the losses between the antenna and rectifier; it does not imply, however, that the entire amount of available power is converted to useful DC power. In fact, RF-to-DC conversion efficiency is the best suitability indicator for the rectifier. Figure 4 shows the RF-to-DC efficiency at different input powers. Simulations of the rectifier are performed in the ADS. Furthermore, the measured results are obtained by powering the rectifier through an RF signal generator and observing the output voltage using a digital multi-meter (DMM). Finally, Equation (4) is used to determine the RF-to-DC efficiency.

4 $$\begin{aligned} \eta (\%) = \dfrac{P_{out}}{P_{in}} \times 100\% = \dfrac{V_{out}^{2}}{R_{L}\times P_{in}}\times 100\% \end{aligned}$$

where $\eta$ is the RF-to-DC efficiency, $P_{out}$ and $P_{in}$ are the respective output and input powers of the rectifier, and $V_{out}$ is the output voltage across the load resistance ($R_{L}$ = 10 $\hbox {K}\Omega$).

**Figure 6:**
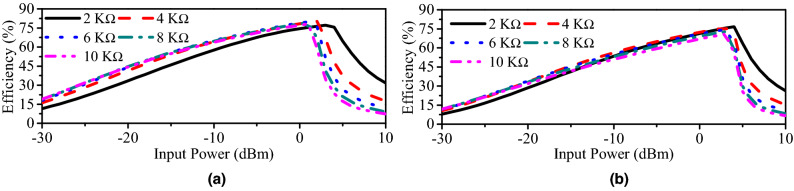
RF-to-DC efficiency at different load resistances. (**a**) At 915 MHz. (**b**) At 2.45 GHz.

**Table 1:** Comparison of the proposed rectifier with state-of-the-art implantable rectifiers.

**Ref.**	**Bands**	Freq. (GHz)	**Topology**	$_{in}$ (dBm)	Load ($\hbox {K}\Omega$)	Eff. (%)
^18^	1	0.915	Full-wave	0	16	50
^19^	1	0.433	Full-wave	11	5	86
^20^	1	1.2	Full-wave	4	–	63
^21^	1	0.673	Half-wave	-	1	40
^22^	1	0.915	Half-wave	−5	2	51.7
^23^	1	0.915	Full-wave	30	8	68.9
^24^	1	0.915	–	−10	3.6	44
^25^	1	2.45	Half-wave	−10	3.3	42
^26^	1	1.9	Full-wave	2	15	82
^27^	1	1.47	Full-wave	2	11	90
This work	2	0.915/ 2.45	Half-wave	0	10	77.02/ 67.04

The simulated and measured RF-to-DC efficiencies of the rectifier against the operating frequency are portrayed in Fig. 4. Evidently, the peak efficiency depends on the available input power. At 0 dBm, the maximum simulated RF-to-DC efficiencies of 77.02% and 67.04% are found at 915 MHz and 2.45 GHz, respectively. For the same input power (0 dBm), the maximum measured conversion efficiencies of 75.1% and 64% are noticed at 0.915 and 2.45 GHz, respectively. It can also be observed that the peak efficiency drops down with decrease in the input power. At the input power of −10 dBm, the measured maximum efficiencies of 58% and 48.2% are observed at the lower- (915 MHz) and higher-frequency band (2.45 GHz), respectively. Similarly, at the input power of −20 dBm, the measured peak conversion efficiencies of 41.3% and 31.2% are noted at the lower- and higher-frequency bands, respectively. Fig. 5 shows the simulated and measured conversion efficiencies of the rectifier as a function of the input power. In the lower-frequency band (0.915 GHz), the simulated (measured) RF-to-DC conversion efficiency of 79.9% (77.3%) is observed at the input power of 1 dBm. Similarly, for the higher-frequency band (2.45 GHz), it has simulated (measured) RF-to-DC efficiency of 72.8% (66.8%) at 3 dBm. In all frequency bands, efficiencies are greater than 30% at a lower input power of −20 dBm. It is worth mentioning that a single frequency and input power signal is generated at a time and the same procedure is adopted for all other frequencies and input powers. Evidently, the proposed rectifier has the ability to harvest signals with very low amounts of power, thus, making it a suitable choice for power-restricted sources. In fact, all simulations and measurements in Figs. 4 and 5 are carried out at a load impedance of 10 $\hbox {K}\Omega$. It is a fact that RF-to-DC efficiency also depends on the load, thus, a series of simulations are performed at varying load resistance, as shown in Fig. 6. Evidently, the peak efficiency is shifted to lower input powers with increasing the load resistance. Besides, a comparison table (Table 1) is added to clearly identify the advantages of the proposed rectifier compared to other implantable rectifiers. It is worth mentioning that none of the implantable rectifiers in the literature can operate in more than one band. Moreover, the RF-to-DC efficiency of the proposed rectifier is better than the majority of rectifiers.

**Figure 7:**
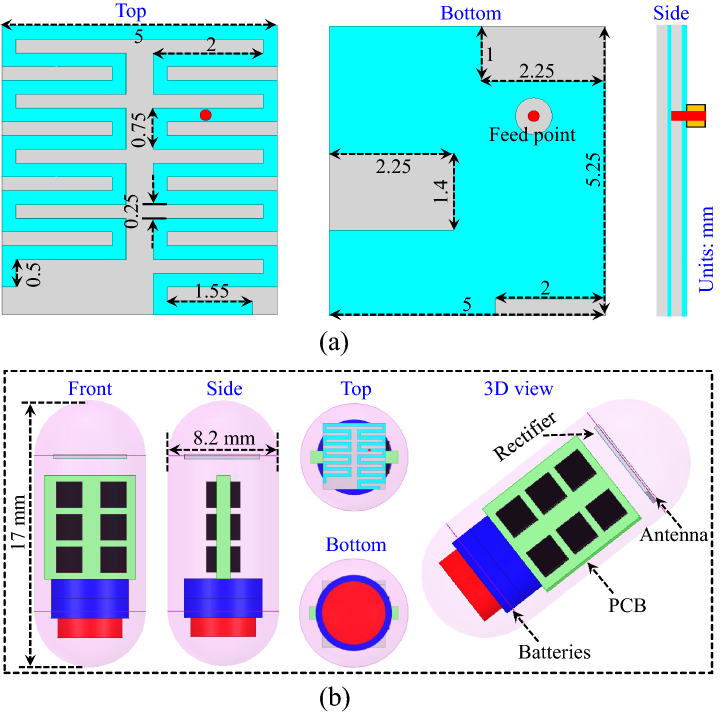
(**a**) Geometry of the proposed dual-band implantable antenna. (**b**) Deep-body device architecture.

## Dual-band implantable antenna

A meandered-resonator based dual-band implantable antenna is designed and optimized in a full-wave electromagnetic simulator (HFSS). The top, bottom, and side views of the proposed antenna are shown in Fig. 7. It can be seen that the meandered-resonator monopole is located on the top layer of the substrate and that the slotted-ground plane is located on the bottom layer of the substrate. The width of the meandered line and the gap between them is 0.25 mm. Moreover, a superstrate is added to facilitate loading effects, thus, achieving miniaturization. A high-permittivity material Rogers RO3010 is used as a substrate and superstrate for size reduction. Moreover, open-ended rectangular slots are added in the ground plane to further achieve miniaturization. The proposed antenna is sourced by a $\hbox {50}\Omega$ coaxial probe. This antenna, along with RF and electronic circuitry, is enclosed in a cylindrical container, as illustrated in Fig. 7b. The cylindrical container is made of alumina material that is 0.2 mm thick. The simulation and optimization of the proposed antenna is performed in the abdomen of the realistic human model in HFSS. The implantable antenna, which is enclosed in a cylindrical implantable device, is implanted 55 mm deep in the abdomen of the human body.

**Figure 8:**
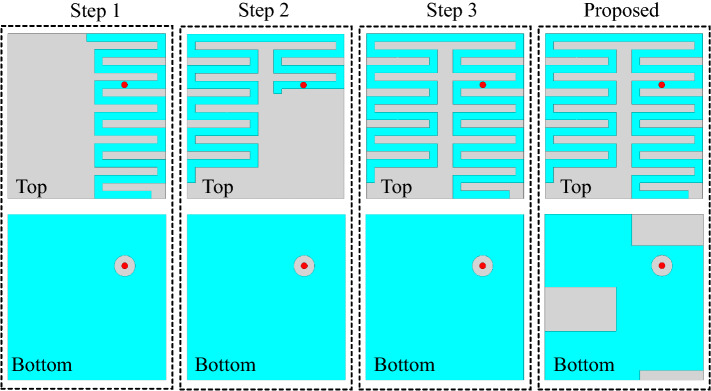
Design iterations of the dual-band implantable antenna.

**Figure 9:**
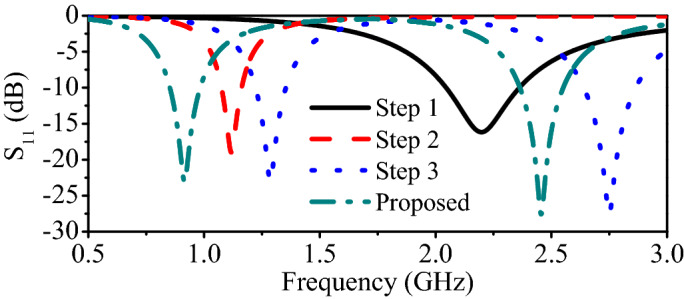
Reflection coefficients of the dual-band implantable antenna in design iterations.

### Design evolution process

The design evolution process of the proposed antenna is shown in Fig. 8, consisting of four steps. Initially, in the first step, a half-wavelength meandered resonator is designed at 2 GHz. It can be observed that the resonance is at 2.2 GHz (Fig. 9). In the second iteration, another stand alone meandered resonator is designed at 0.915 GHz. At this stage, the resonator resonates at 1.15 GHz. The resonant frequency of the resonator is higher than expected because of the less-effective region of the resonator. Furthermore, in the third step, both individually designed resonators (from the first and second step) are co-designed to constitute a dual-band antenna. It can be noted that the antenna resonates at 1.27 GHz and 2.65 GHz. Finally, in the last iteration (the proposed antenna), three open-ended slots are added to the ground plane to reduce the overall size of the antenna. As a result, the resonant frequencies are shifted to 0.915 GHz and 2.45 GHz, as shown in Fig. 9. In fact, the open-ended slots at the ground plane induce additional capacitance. Therefore, the resonant frequencies are shifted to the lower-frequency side. Moreover, the addition of capacitive slots on the miniaturization process can be easily understood using slow-wave theory [Equation (5)]^35^. It can be observed that the resonant frequency is mainly dependent on the capacitance and inductance of the antenna. In fact, both capacitance and inductance have inverse relationships with the resonant frequency of the antenna. Thus, increasing the value of capacitance shifts the resonant frequency to the lower-frequency side.

5 $$\begin{aligned} v_{p} = \dfrac{c}{\lambda _{g}\sqrt{\epsilon _{eff}}} = \dfrac{1}{\sqrt{L_{a}C_{a}}} = f_{r} \end{aligned}$$

where $f_{r}$ is the resonant frequency of the antenna, $v_{p}$ is the propagation speed, $\epsilon _{eff}$ is the permittivity of the substrate, $\lambda _{g}$ is the wavelength at $f_{r}$, $L_{a}$ is the inductance of the antenna, and $C_{a}$ is the capacitance of the antenna.

**Figure 10:**
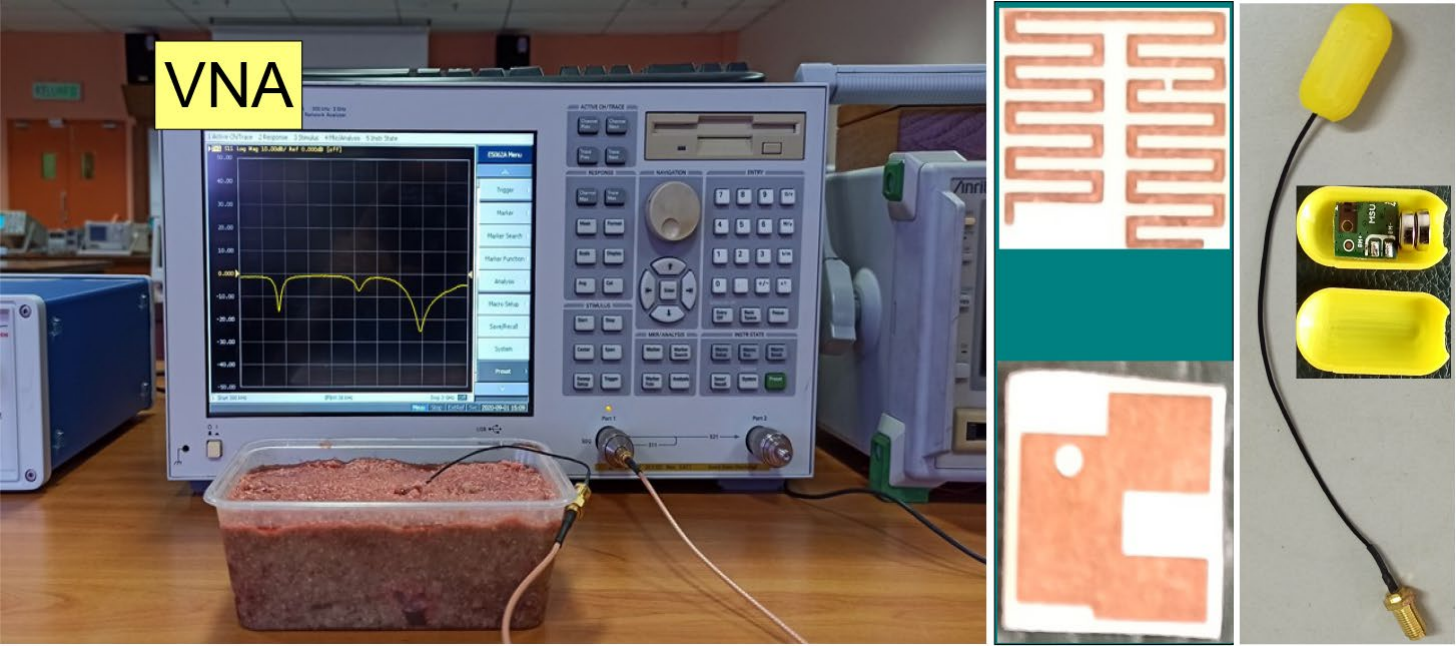
Reflection coefficient measurement setup of the proposed antenna.

**Figure 11:**
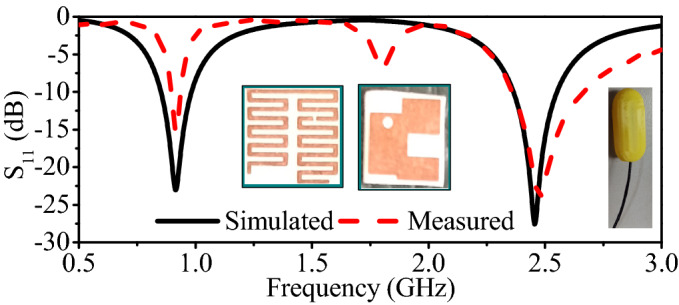
Simulated and measured reflection coefficient of the implantable antenna.

### Simulation and experimental results

**Figure 12:**
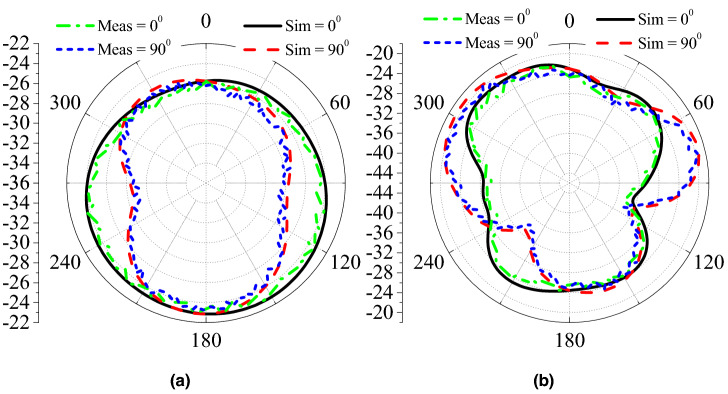
Radiation patterns of the antenna at (**a**) 915 MHz and (**b**) 2.45 GHz.

The proposed antenna is simulated in the abdomen of the realistic human body, which resonates at 0.915 GHz and 2.45 GHz with a reflection coefficient lower than −20 dB. It can be observed that the antenna covers the desired ISM bands (0.915 GHz and 2.45 GHz) and has a FBW of 14.5% and 8.2% at 0.915 GHz and 2.45 GHz, respectively. To verify the simulated results, the proposed antenna is designed on a Rogers RO3010 substrate. The meandered-resonator lines and slotted ground is designed using chemical etching. Moreover, the cylindrical container is printed using three-dimensional (3D) printing technology. The proposed antenna, RF, and electronic circuitry are placed inside the container and sealed with epoxy, as shown in Fig. 10. Then, the measurements are performed by embedding the capsule device inside a box containing minced pork meat. The reflection coefficient measurements are performed by connecting the antenna with a vector network analyzer (VNA) model E5062A of Agilent Technologies. The measured reflection coefficient is compared with the simulated one in Fig. 11. It can be observed that the measured reflection coefficient has a resonant frequency of 0.916 GHz (lower band) and 2.49 GHz (higher band). Moreover, the measured FBWs are 8.1% and 11.4% at the lower and higher bands, respectively. Figure 12 shows E-plane and H-plane radiation patterns of the implantable antenna at 0.915 GHz and 2.45 GHz. The radiation patterns at both frequency bands are quasi-omnidirectional, which is highly desirable for a moving implant to harvest the available power from all directions. Moreover, it has peak gains of −22.1 dBi and −19.6 dBi at 0.915 GHz and 2.45 GHz, respectively.

**Figure 13:**
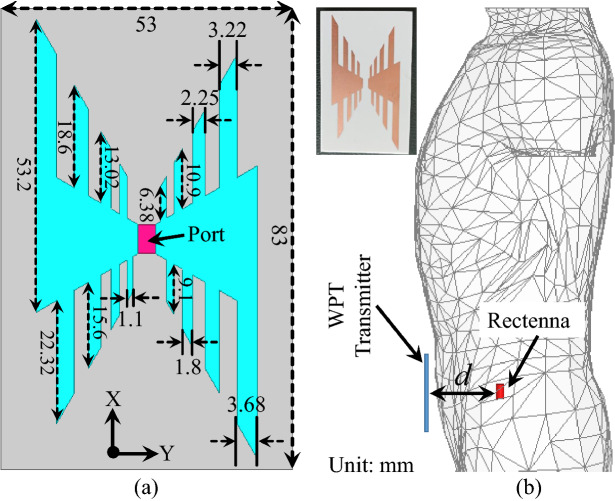
(**a**) Schematic diagram of WPT transmitter, and (**b**) Wireless powering setup of the implant ($d = 60$ mm).

## Wireless power transfer transmitter

**Figure 14:**
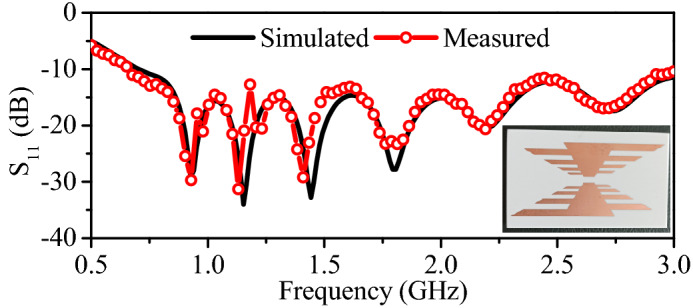
Simulated and measured reflection coefficient of the WPT transmitter.

A WPT transmitter is a fundamental part for transferring RF power to deeply implanted BIDs. Figure 13a shows the geometry of the proposed log-periodic WPT transmitter. A log-periodic antenna is chosen as a WPT transmitter because of its directional beam and wide bandwidth. The radiating structures of the log-periodic antenna are designed on a 1.28 mm thick Rogers RO3010 substrate. The overall dimensions of the WPT transmitter are $83\times 53\times 1.28$ $\hbox {mm}^{3}$. A $50\Omega$ lumped excitation scheme is used in the simulation. During the simulations, the WPT Transmitter is placed at a distance of 5 mm from the skin of the abdomen, as shown in Fig. 13b. The WPT has wide bandwidth, covering the frequency band from 0.62 GHz to 3 GHz, as shown in Fig. 14. The $\hbox {S}_{11}$ measurements of the WPT transmitter are carried out by placing it on side of the minced pork meat and connecting it with the network analyzer. The measured results show a good level of agreement with the simulated ones.

**Table 2:** Comparison of the proposed WPT system with state-of-the-art systems.

**Ref.**	^19^	^21^	^22^	^23^	^24^	^25^	^26^	^27^	^36^	This work
**WPT System**										
WPT bands	One	One	One	One	One	One	One	One	One	Two
Frequency (GHz)	0.433	0.655	0.915	0.915	0.910	2.45	1.9	1.470	1.5	0.915, 2.45
WPT distance (mm)	500	500	500	1500	20	500	20	50	55	60
Transmitted power (mW)	1000	10	317	–	–	1000	1000	1000	1000	1000
Received power (mW)	0.15	–	–	–	–	–	2.5	6.7	4.73	8.3, 13
PTE (%)	0.015	0.06	0.01	0.0001	0.01	0.007	0.25	0.67	0.473	0.83, 1.3
**Implantable Antenna**										
Size ($\hbox {mm}^{2}$)	$10\times 10$	$\pi$ $\times 5^{2}$	$16\times 14$	$\pi$ $\times 5.4^{2}$	$13\times 13$	$4\times 8$	$5.6\times 6$	$\pi$ $\times 3.5^{2}$	$20.5\times 31$	$5\times 5.25$
Frequency (GHz)	0.433, 2.45	0.675	0.915	0.915	0.915	2.45	0.915, 1.9	0.403, 0.915, 1.470, 2.45	0.403, 1.5, 0.915, 2.45	0.915, 2.45
Bandwidth (%)	26, 2.9	2.2	5.7	2.7	3.8	4.1	9.83, 27.9	–	–	14.5, 8.2
Gain (dBi)	−11, −15	−15.37	−24.3	−23.2	−29	−19	−26.8, −18.8	−34, −19.6, −28.2, −22.4	~ −34.6	−22.1, −19.6

**Figure 15:**
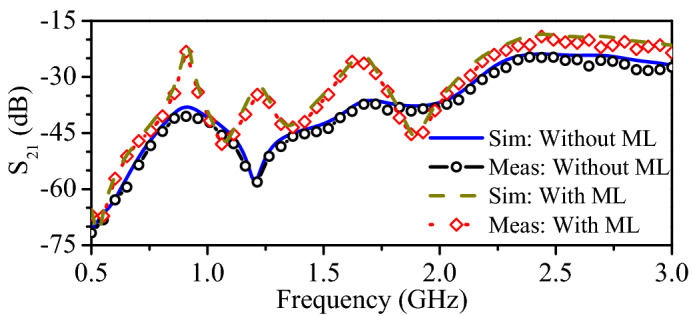
Transmission coefficient ($\hbox {S}_{21}$) of the WPT system with and without the matching layer (ML).

## Results and discussion

**Figure 16:**
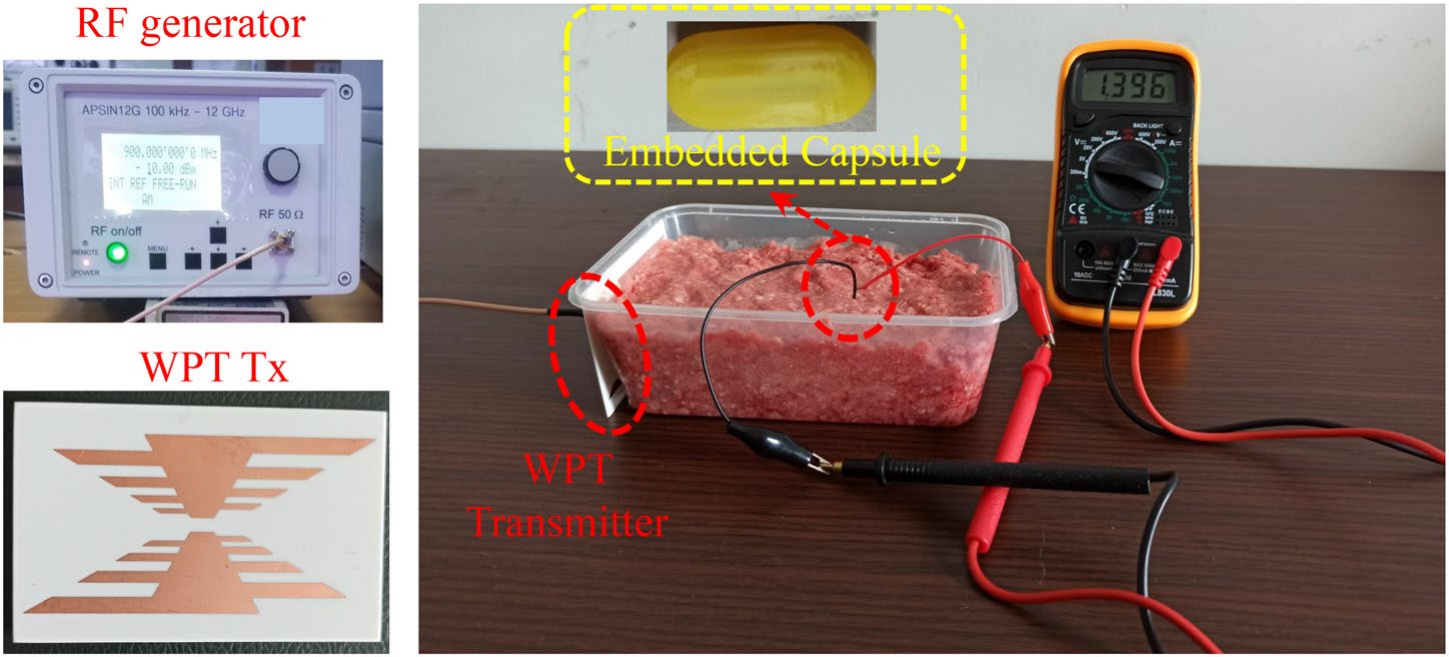
Measurement setup of the rectenna. An RF signal generator is used to source the WPT Tx and output (in terms of voltage) of the rectenna is observed using DMM.

Initially, the dual-band rectifier, dual-band implantable antenna, and wideband WPT transmitter are individually designed and experimentally validated. In this section, all these parts are integrated to wirelessly power the implant. In fact, the proposed dual-band rectifier is integrated with the dual-band implantable antenna to design the dual-band rectenna. Then, the rectenna and the other electronic and RF components are packed inside a capsule device. Next, the capsule device is implanted in the intestine of a realistic human model, as shown in Fig. 13b. Additionally, the proposed WPT transmitter is placed at a distance of 5 mm from the abdomen of the human model. To determine the PTE of the system, the proposed WPT transmitter is considered as a source and implantable antenna is considered as a receiving unit. The transmission coefficient ($\hbox {S}_{21}$) of the proposed WPT system is shown in Fig. 15. The $\hbox {S}_{21}$ of the WPT system is −37.9 dB and −23.7 dB at 0.915 GHz and 2.45 GHz, respectively. In fact, the WPT transmitter (outside a human body) operates at different medium than the implantable antenna (inside a human body). Therefore, more power is radiated back from the human body due to the dielectric mismatch. These mismatches can be reduce using high-permittivity matching layers^27^, metasurfaces and metamaterials^37–40^, parasitic patches^25^, and conformal surfaces^41^. In order to minimize such a mismatch, a matching layer of high dielectric constant can be used^27,36^. In this work, a matching layer ($40\times 40\times 0.1$ $\hbox {mm}^{3}$) of Rogers 3010 ($\epsilon _{r}$ = 10.2) is used. As a result, the $\hbox {S}_{21}$ of the WPT system is observed as −20.8 and −18.6 dB at 0.915 and 2.45 GHz, respectively. Consequently, the PTE of the WPT system is improved from 0.07% and 0.43% to 0.83% and 1.3% at 0.915 and 2.45 GHz, respectively. Furthermore, the simulated $\hbox {S}_{21}$ results are verified through practical measurements. In measurements, the WPT transmitter is connected to one port of the VNA and the implantable antenna is connected to the second port of the VNA. The practical results agree well with the simulated ones. To verify the wireless powering and RF-to-DC conversion efficiency of the implant, the WPT transmitter is connected to an RF signal generator and output of the rectifier is connected to a digital multi-meter. The capsule implant is placed in the center of a box containing minced pork meat, and the WPT transmitter is placed at a distance of 60 mm from the implant, as shown in Fig. 16.

**Figure 17:**
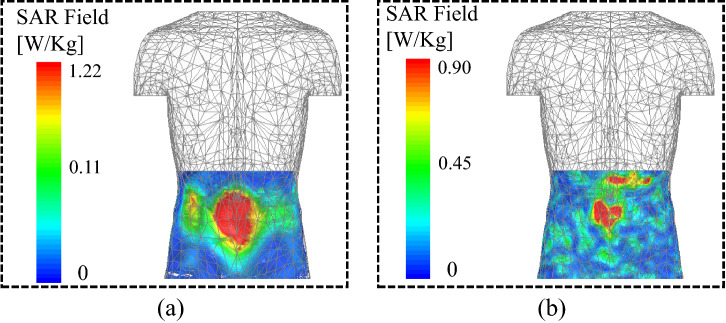
Specific absorption rate of the WPT system at (**a**) 0.915 GHz, and (**b**) 2.45 GHz.

The specific absorption rate (SAR) analysis is performed to ensure patient safety during wireless powering of the capsule implant. The SAR analysis is performed by considering 1-g of tissue and an input power of 1 W (30 dBm), as illustrated in Fig. 17. The capsule device is placed in the intestine of the human body and the WPT transmitter is placed at a distance of 5 mm from the abdomen. At an input power of 1 W, the peak SAR values of 1.22 and 0.90 W/Kg are observed at 0.915 and 2.45 GHz, respectively. The SAR values in both bands are lower than the standard provided by the Institute of Electrical and Electronics Engineers (IEEE). The standard limit for 1-g tissue is 1.6 W/Kg for an input power of 1 W; thus, the proposed WPT system is suitable for the wireless powering of the biomedical implants using 0.915 and 2.45 GHz frequency bands.

Table 2 shows a comprehensive comparison of the proposed WPT system with state-of-the-art WPT systems. The unique advantage of the proposed WPT system is that it can efficiently rectify two RF signals, while all other WPT systems can only harvest one RF signal. Other advantages of the proposed system include higher PTE in both bands, the compact dimensions of the implantable antenna, and the implantable antenna’s higher gains. Moreover, the proposed WPT transmitter has a wideband, which can be used as a transmitter for any system operating between 0.62 and 3 GHz.

## Conclusion

In this paper, a dual-band implantable rectenna has been simulated and practically validated for recharging and operating medical implants at 0.915 GHz and 2.45 GHz. The implantable antenna is miniaturized using a meandered resonator and by etching open-ended slots on the ground plane. The implantable antenna has compact dimensions with FBWs of 14.5% and 8.2% at 0.915 GHz and 2.45 GHz, respectively. Moreover, omni-directional radiation patterns with peak gains of −22.1 dBi and −19.6 dBi are observed at 0.915 GHz and 2.45 GHz, respectively. Similarly, a dual-band rectifier is simulated and practically demonstrated at 0.915 GHz and 2.45 GHz. The dual-band implantable antenna is integrated with the dual-band rectifier to form a dual-band rectenna. The rectenna, electronic and RF components are packed inside a capsule device and implanted in the intestine of the realistic human model. At an input power of 0 dBm, the rectifier has RF-to-DC conversion efficiency of 77.02% and 67.04% at 0.915 GHz and 2.45 GHz, respectively. A wideband WPT transmitter is also designed, which operates between 0.62 and 3 GHz, to provide an RF signal to the rectenna. After each component of the WPT system is individually simulated and practically validated, finally, as a proof, the WPT transmitter is sourced with an RF signal generator and the rectenna is placed inside minced pork meat and the received voltage is observed on the DMM. Using a matching layer, the PTE of the WPT system is improved from 0.07% and 0.43% to 0.83% and 1.3% at 0.915 GHz and 2.45 GHz, respectively. Furthermore, it has a SAR at both frequency bands, where the peak SAR is 1.22 W/Kg at 0.915 GHz and 0.90 W/Kg at 2.45 GHz. The proposed implantable rectenna shows good performance in terms of its dual-band operation, human safety analysis, high RF-to-DC conversion efficiency, and compact dimensions. Moreover, this is the first implantable rectenna that performs dual-band RF signal rectification.

## Data Availability

All data generated or analysed during this study are included in this published article.
